# Accessing Antenatal Care (ANC) services during the COVID-19 first wave: insights into decision-making in rural India

**DOI:** 10.1186/s12978-022-01446-2

**Published:** 2022-07-08

**Authors:** Shweta Bankar, Deepika Ghosh

**Affiliations:** Centre for Social and Behaviour Change, Ashoka University, Haryana, India

**Keywords:** Antenatal care services, Behavioral principles, Childbirth, COVID-19, India, Pregnant women

## Abstract

**Background:**

Antenatal care (ANC) services are a prime focus of the Government of India's National Health Mission (NHM), of which a key pillar is the promotion of maternal and child health. To ensure uninterrupted service delivery at the last mile, a cadre of Frontline Health Workers (FLHWs) has been appointed and health centres established at the village level. However, the onset of the COVID-19 pandemic and the nationwide lockdown from late March to June 2020 impacted pregnant women's access to institutional antenatal care services.

**Methods:**

Using a qualitative research design, data was collected through 12 in-depth interviews (IDIs) with pregnant women and 17 IDIs with frontline health workers in the selected six districts under study. The narratives were analysed using inductive coding in Atlas.ti.

**Results:**

During the first wave of the COVID-19 pandemic, pregnant women, most of whom belonged to poor and marginalised groups, were left with limited access to health centres and FLHWs. Respondents from the study areas of rural Jharkhand, Madhya Pradesh and Uttar Pradesh extensively reported concerns stemming from the lockdown that influenced their decision to access ANC services. These included anxieties around meeting their families' daily needs due to a loss of livelihood (in particular, abject food insecurity), inability to access healthcare, and a sense of mistrust in public health systems and functionaries. All of these, coupled with the real threat to health posed by COVID-19, disrupted their plans for pregnancy and delivery, further compunding the risk to their health and wellbeing.

**Conclusion:**

This study identified several social, behavioural and structural facets of the communities that contributed to the confusion, anxiety and helplessness experienced during the COVID-19 first wave by both groups, viz. pregnant women and FLHWs. In planning and implementing initiaves to ensure the delivery and uptake of ANC services in this and similar contexts during times of crisis, these facets must be considered.

## Plain language summary

This article highlights the status of ANC services during the nationwide lockdown imposed in the first wave of the COVID-19 pandemic in the Indian states of Jharkhand, Madhya Pradesh and Uttar Pradesh. Data was collected through 12 in-depth interviews with pregnant women and 17 in-depth interviews with frontline health workers. Findings suggest that pregnant women were aware of the unprecedented threat of COVID-19 and recommended protective measures through trusted sources of information, including their local Accredited Social Health Activist (ASHA). However, both pregnant women and FLHWs believed that the information they received on the health risks of the pandemic and strategies to address these was inadequate. Temporary suspension of health services in the rural countryside meant that pregnant women could not track the foetus's health status, resulting in confusion and distress. Limited or no interaction with FLHWs, coupled with a reported lack of attention to conditions unrelated to COVID-19 and discrimination at healthcare institutions increased the uncertainty around institutional deliveries. This was further heightened by the loss of livelihoods due to the shutdown of businesses during the lockdown, as the respondents could not turn to private hospitals for childbirth. This resulted in the collapse of the trust of pregnant women and their families in the public healthcare system. To bridge this gap and alleviate the sense of mistrust the pandemic has created in its end-users, strategies to improve the utilisation of health services should respond to the barriers identified in this study.

## Background

Maternal mortality is a priority area under the United Nations’ Sustainable Development Goal (SDG) 3, which aims to ensure health and promote well-being for people of all ages [1]. The Government of India is committed to lowering the Maternal Mortality Ratio (MMR) from 130 (maternal deaths per 1,00,000 live births) in 2016 to 70 by 2030 [2]. Taking into account the extensive challenges in the provision of comprehensive approach to reduction of MMR, ANC services and the access to safe delivered especially in rural areas were strengthened. The Government of India (GoI) has designed and implemented interventions to improve the reach and quality of ANC services, as well as augment demand for these services. Programs implemented in the last 15 years include the National Rural Health Mission (2005) [3], the National Urban Health Mission (2008) [4], and the Reproductive, Maternal, New-born, Child, and Adolescent Health (RMNCH + A) Strategy, introduced in 2013 [5]. In the last 10 years there has been a marked improvement in the uptake of these services, given the government’s efforts and sustained engagement of frontline health workers [6–8]. Most recently, in 2016–18, India’s MMR was down to 113 per 1,00,000 live births, yet there is a long way to go [9]. The percentage of pregnant women attending at least four ANC contacts increased from 43.9 (NFHS-3) to 58.6% (NFHS-4) [10]. As per NFHS-5 data released for 22 Indian states and UTs, the percentage of women availing of ANC services in the first trimester has increased in 18 states/ UTs, and the percentage of women completing the recommended minimum of four ANC visits has increased in 11 states/ UTs in the years since NFHS-4 [11].

In India, official guidance for pregnant women recommends a minimum of four antenatal care contacts during pregnancy, at specified intervals. However, during the COVID-19 pandemic, pregnant women were advised not to visit sites of ANC check-ups in the months of April and May 2020. The Indian Council for Medical Research (ICMR) and National Institute for Research in Reproductive Health (NIRRH) issued communication in the initial days of the lockdown that detailed the updated protocol for childbirth, such as reservation of obstetrics units for confirmed COVID-19 positive cases; and ensuring that the pregnant woman, Healthcare Workers (HCWs) and birth partner all wear PPE during labour and delivery [12]. However, there was a gap in frontline health workers’ awareness and comprehension of these guidelines, and this led to an atmosphere of confusion and fear at the grassroots [13]. An overall lack of preparedness in the form of PPE kits and infrastructure, administrative apathy, persistent socio-economic inequalities and resulting discrimination, and apathy towards returning migrants were some of barriers in accessing ANC services as the lockdown was eased [13, 14].

Besides the unavailability of check-up facilities, there were other services, chiefly home visits to pregnant women and distribution of Take-Home Ration (THR) that also proved inaccessible. Supply-side barriers to access included disruption in the transportation of supplements like iron and folic acid (IFA), zinc and calcium tablets, burden on health workers of COVID-19-related tasks, which reduced their availability for maternal healthcare service delivery [15]. On the demand side, key barriers included inaccurate or incomplete information about service provision, fear of contracting COVID-19 due to stepping out of the home, widespread loss of livelihoods, restrictions on mobility, etc. [16, 17]. Services were restored by 68–90% of FLHWs in July 2020, and among them, immunisation was the first to resume followed by home visits by the FLHW, and distribution of supplementary nutrition [18].

Regional studies described a high level of awareness among pregnant women around precautionary behaviours to be adopted during COVID-19 [19, 20]. However, studies also brought to light pregnant women’s anxieties around securing food and shelter in the absence of paid work, the suffering of their children due to school closure and confinement in the home, and lack of social support (among those belonging to ethnic minorities and other marginalised groups) [21]. Even during the unlocking process, uptake of most ANC services did not improve due to constraints on travelling (such as the limited functioning of public transport, apprehension around using public transport due to chances of contracting infection, and lack of access to private vehicles for transportation to sites of ANC check-ups); non-availability of services; a general fear of contracting the disease when outdoors, which was heightened at the prospect of interface with healthcare functionaries and these influenced the decisions about the uptake of the services [18, 21].

Lockdown in India aggravated women’s perinatal anxiety, and they felt low confidence in giving birth during the pandemic. They were no longer able to avail of healthcare services as per the planned cadence of ANC check-ups, and often had to forego attending them due to lack of reliable means of transportation, familial pressure to self-isolate, low value perception of ANC in the eyes of key family members, fear of contracting the virus, vulnerability to complications in pregnancy, and risk of transmitting COVID-19 to the fetus, if they contracted the illness [13, 18]. Due to the uncertainty around the wellbeing of their families, and the proliferation of unreliable sources of information, pregnant women were left anxious and impaired in their capacity to prepare for childbirth, which further exacerbated their distress [21].

Given the outcomes of the situation arising from the lockdown in India in the first COVID-19 phase, both at the systemic level and at the personal level, we understand that the decisions around accessing non-COVID-19 health services were highly biased. This had a significant impact on the access and utilisation of key services like ANC and Safe Childbirth. This paper highlights the factors that influenced the uptake of ANC services and the institutional deliveries at the time of the lockdown. We present our findings from a sample of 12 pregnant women and 18 Frontline Health Workers in rural India. The insights are drawn from the in-depth interviews conducted with the respondents.

## Methods

This study is reported as per the Consolidated Criteria for Reporting Qualitative Research (COREQ) [22].

### Aim, design and setting of the study

This qualitative research study was carried out by the Center for Social and Behavior Change (CSBC), Ashoka University, and funded by the Bill and Melinda Gates Foundation (BMGF). Jharkhand, Madhya Pradesh and Uttar Pradesh, India were selected as study locations, due to CSBC’s focus on these states as part of its ongoing partnership with the National Institute for Transforming India (NITI) Aayog to improve health outcomes in these states. Two districts in each of these states were selected for this study, based on the presence of development partner organisations in the districts. While preference was given to Aspirational Districts, the final selection of districts was not limited to these.

This paper draws on data collected as part of a large-scale exploratory research study led by CSBC to understand the landscape of health and nutrition services in the times of COVID-19 in March 2020, and a phased re-opening of the same in the selected states thereafter. The data was collected over a period of three months to grasp the status of services and the challenges faced by end users and service providers during the lockdown and Unlock 1.0. Ethical review and clearance were provided by the Institutional Review Board of Ashoka University. A qualitative research design was adopted to study diverse aspects of ANC services and institutional deliveries in these times.

In this paper, we share findings from 12 in-depth telephonic interviews conducted with pregnant women and 17 in-depth interviews with frontline health workers. Pregnant women’s interviews explored themes of knowledge, awareness and practices in the times of COVID-19 pandemic; fears and anxieties around pregnancy; the planning of childbirth and their needs for support (including from frontline health workers); and the challenges anticipated during childbirth. In-depth telephonic interviews with frontline health workers focussed on the change in their work schedule and responsibilities owing to the pandmeic, the institutional emphasis on preventing and managing COVID-19, and its effect on the community, especially pregnant women.

The interviews were conducted by the qualitative research team at CSBC (including the authors of this paper) and trained field enumerators, and lasted approximately 45–60 min. Each of the interview sessions was followed by a detailed debriefing. Interview guides were adapted to the categories of respondents and comprised questions that were open-ended in nature, followed by probes. While some questions could be answered just in the affirmative or negative, interviewers actively utilised probes to encourage further discussion around responses.

### Sample characteristics

Convenience sampling was undertaken of pregnant women, while ensuring representation from three distinct sections of the community—general population, scheduled caste/tribes and returning migrants. Similarly, FLHWs were conveniently sampled, representing one Auxiliary Nurse Midwife (ANM), Accredited Social Health Activist (ASHA) worker, and Anganwadi worker (AWW) for each district. With the help of a matrix, each of the FLHWs were represented by one respondent from within the district central place and two from villages located far from the district centre. Table 1 outlines the inclusion criteria and distribution of the sample across the states.

Interviews were conducted in Hindi, Kho, and Bhojpuri, at a time convenient for respondents and enumerators. Oral consent was obtained from each respondent before the interviews commenced. Care was taken to ensure that the telephonic engagement took place in a safe environment and maintained participants’ confidentiality. All interviews were audiotaped, transcribed and translated verbatim into English by an external agency, and these translations were verified by interviewers to ensure accuracy.

### Data analysis

The methods of thematic and narrative analyses [23] were adopted for analysing the data collected. The team followed a step-wise process of familiarising themselves with the data, identifying a thematic framework and developing a coding frame. In the preliminary stages, the data from notes taken by enumerators and CSBC researchers was thematically analysed, in order to draw high-level insights through a deductive approach. Further, the data was subjected to a thorough qualitative analysis with inductive coding, using the software Atlas.ti 9.0.

### Findings

The findings highlight the different behavioural, financial, social and infrastructural factors that influenced pregnant women’s and their families decision on the uptake of the ANC services and planning of safe deliveries.

### Pregnant women’s knowledge and risk perception

Pregnant women were aware about the COVID-19 situation in the country, its symptoms and the precautionary measures they had to engage in. When asked if they knew of any other diseases that compared to COVID-19 in severity, the majority of respondents expressed that COVID-19 was the most formidable disease they had ever heard of, due to its rapid spread, high rate of fatality, and the fact that no cure had been found for the disease.

*“There are other dangerous diseases, but there are solutions… there are medicines (for those diseases). But there is no treatment for this corona (sic), and that is why we are afraid.”* (Pregnant Woman, District Khunti, Jharkhand)

A minority across the study areas said that this was just like the dozens of other dangerous diseases that were common, such as those borne by contaminated water. Their thinking was influenced by the high incidence of water-borne diseases in their community. They feared that the concentration of efforts on the COVID-19 situation would delay timely and appropriate treatment of other illnesses and conditions, which may, in the absence of appropriate medical attention, prove fatal just like COVID-19.

There was heightened awareness of COVID-19 being especially dangerous for pregnant women, as respondents had been told so by ASHAs. Some pregnant women across study areas reported receiving official text messages advising them to take special protective measures as they were especially vulnerable at this time. Respondents were emphatic that COVID-19 would adversely impact their health and the fetus’ development, but did not have any specific information on how this would happen. FLHWs confirmed this messaging and reported spreading these messages over phone or home visits.

*“The ASHA told us “If you stay at home, you will stay safe. Don’t go to the hospital, (as) many different kinds of people are going there. It is unsafe for you, and for your child as well… that’s why you should stay in your houses.”* (Pregnant Woman, District Lalitpur, Uttar Pradesh)

Pregnant women were aware of protective measures such as avoiding going out of their homes or consuming outside food, covering their faces with masks, washing hands with soap and water, and maintaining social distance. They were made aware of these, along with routine pregnancy care guidance (such as what food items to consume, and how much rest to get each day) by the FLHWs in their community. During lockdown, FLHWs typically communicated with them over phone calls, and sometimes by visiting them at home. During home visits, FLHWs made sure of no-contact and followed all the other COVID-19 protocols. At the time of the interviews, in the early months of the COVID-19 lockdown in India, the effects of COVID-19 on pregnant women were unknown, and hence there was no official guidance on the same as reported by an ASHA and reciprocated by other FLHWs across the study geographies.

*“We have been told to inform pregnant women about the COVID-19 situation and that they are highly vulnerable to the infection but we don’t know how. Pregnant women ask us about this and we have no answers.”* (ASHA, District Chattarpur, Madhya Pradesh)

The respondents’ other key sources of information about the virus and pandemic included their mobile phones (via SMS, Government of India mandated COVID-19 advisory caller tune, and internet for those who owned smartphones), television and word of mouth.

### Anxieties around the pandemic

Almost all pregnant women reported being acutely worried about making ends meet during the pandemic and this negatively impacted the nutrition of the family and their special nutrition needs given their pregnancy. Owing to the lockdown, there was widespread loss of livelihoods, and this led to abject insecurity around access to food. All respondents reported that their husbands were at home all day, without any work that would enable them to earn wages. With their children bound to the home, respondents felt that their education was suffering in the process. Concerns around their health during pregnancy, such as consuming a nutritious, well-balanced diet, were secondary to the daily anxieties of putting food on the table for their families. This was especially true for respondents who did not have ration cards in the family and hence could not take advantage of the government’s relief schemes. Even those who received rice under the government scheme (Pradhan Mantri Garib Kalyan Anna Yojana) said that it was grossly inadequate to meet the needs of the family.

While some pregnant women received supplementary nutrition for their children, others whose children were over 5 years of age and no longer eligible for supplementary nutrition remarked that this further built up pressure on them to feed their children. Since respondents were pregnant, they were not in the physical condition to work and earn money for the household, which only compounded their stress. Some pregnant women were inconsolable as they narrated their hardships, having resorted to picking shrubs and plants from around their homes and cooking them for the children in their family, or eating roti with salt for their meals.

*“We get 5 kg of rice in a month for each person, which is not sufficient for my child, husband and me. So I search the bushes around my home for anything that can be eaten. I face a lot of problems, and feel very disheartened.”* (Pregnant Woman, West Singhbhum, Jharkhand)

Additionally, there was a recognition that the pandemic-induced lockdown had made their experience of pregnancy very difficult, in terms of procuring appropriate foods, medicines, etc. A small set of first-time expecting mothers reported feeling especially demoralised, as they did not have relatives nearby to guide them and could not access ANC services (as well as guidance from health workers) during the lockdown. This contributed to a feeling of helplessness as these women were facing dire material circumstances, including food insecurity, and were unable to do anything to remedy the situation.

### Access to healthcare services

Access to healthcare facilities was suspended during the lockdown and when the process of unlocking began, pregnant women faced structural and behavioural challenges. Pregnant women are recommended to visit ANMs four times over the course of their pregnancy at a Primary Health Centre (PHC), Anganwadi Centre (AWC) or monthly Village Health, Sanitation and Nutrition Day (VHSND), where they receive a host of services including physical examination, injections/immunization and supplements, investigations like blood tests and ultrasounds, as well as counselling on appropriate care. Respondents expressed their fears about availing these services at health centres even after taking due precautions, since they could contract coronavirus from meeting others.

*“I am afraid that it is possible for a pregnant woman to get corona easily, that's why we are… scared when we go to the hospital.”* (Pregnant Woman, District Khunti, Jharkhand)

They were also especially apprehensive of seeking healthcare services when it entailed travelling, since public transport was shut down and ambulances may increase their exposure to the virus (as it was suspected that they were being used to transport COVID-positive persons). This was further confirmed by FLHWs as in the following quote,

*“We do not encourage pregnant women to use the ambulances which were actually for their use but are now used to transport the COVID-19 patients due to shortage of vehicles. The ASHAs are instructed to bring them in private transport and the fare gets reimbursed…. But I don't know what they (ASHAs) are doing.”* (ANM, District Faizabad, Uttar Pradesh)

In several cases, the pregnant women were discouraged from visiting health centres by FLHWs themselves due to the spread of COVID, instead being recommended to seek phone consultation. In case ANC services were not being delivered at AWCs, pregnant women had no option but to turn to higher level public health centres. Their fears around COVID-19 were compounded by their belief that hospital staff would treat them poorly, due to negative past experiences with institutional healthcare, and rumours of the discrimination and ill-treatment in the community.

Despite these apprehensions, there was angst at not being able to access ANC services during lockdown, as there was no way to know if the pregnancy was progressing normally and if the fetus was developing as it should.

*“Doctors were saying that ultrasound is to be done twice… then only they come to know about the status of the baby and health of the mother. How can this be known if we don’t consult the doctor?”* (Pregnant Woman, District Khunti, Jharkhand)

Pregnant women returned to AWCs and hospitals for check-ups once the lockdown was eased, since they felt it was essential for the health of their child. However, there was no mention of wanting to attend ANC contacts for the benefits to their own health- likely because during pregnancy, the health of the baby is of utmost importance, while the health of the mother is important to the extent that it impacts the baby's health, which leads to a devaluation of the latter.

### Service delivery by healthcare functionaries

In some districts of Uttar Pradesh, pregnant women reported not being able to attend any ANC check-ups due to the lockdown.

*“Due to the lockdown... when we went (to the hospital), there were no doctors... and no one was doing checkups, so we came back…”* (Pregnant Woman, District Lalitpur, Uttar Pradesh)

However, some pregnant women reported that ANC check-ups continued as normal, except that they received vaccination, Mother and Child Protection (MCP) card and medicines (IFA, calcium, etc.) from a distance, and were guided by the ANM to wear a mask or face covering. Additionally, they were called into the AWC in small numbers for vaccination to prevent crowing. In rare occasions, pregnant women reported being given sanitiser to disinfect their hands as well prior to the meeting.

*“There isn’t permission for a lot of people, so two women enter (the health centre) and get the vaccinations.”* (Pregnant Woman, District Chitrakoot, Madhya Pradesh)

Of these women, some felt slighted by this new manner of service delivery, saying that they had to ask permission before entering the health centre, were told to sit at a distance from the ANM (which they felt was unnecessary), and were dealt with hurriedly. Some even said that while they were treated as usual, the FLHWs’ treatment of lower caste beneficiaries was different.

*“Their (ANM’s) behaviour towards the Ahirwal family was quite indifferent. Untouchability is a big problem.”* (Pregnant Woman, District Chhatarpur, Madhya Pradesh)

In the phase of unlock 1 (early May 2020), ANC services were being delivered by ANMs in their most rudimentary form at the AWC—among them, immunisation and physical examination were prioritised. IFA supplementation delivery varied from district to district—while some pregnant women reported receiving their share of IFA tablets from the AWC, others said that the AWC was shut and hence there was no supply of tablets, while still others said that they had not received any tablets from the AWC since the start of the pandemic but were yet to finish their stock from earlier. In many places, blood and urine tests and weight check could not be accessed because of non-functioning infrastructure or because FLHWs did not have time to administer them.

*“I was thinking that (the strip of IFA tablets distributed) may have been touched by many people and may carry the virus when I returned from the hospital. But later I thought that it is not good to not eat the medicine, (and) I removed the negativity (sic).”* (Pregnant Woman, District Khunti, Jharkhand)

According to FLHWs, their engagement in the COVID-19 related tasks allowed very little time to cater to the ANC needs of the pregnant women. They promoted the use of tele-consulting as it saved time and effort and was the safest means in the given situation. With most of the services suspended, they could not cater to their ANC needs and the disruption of supply of the IFA, calcium and zinc tablets caused shortage in supply.

Several pregnant women in West Singhbhum (Jharkhand) and Chhatarpur (Uttar Pradesh) reported only able to access ANC services and guidance at government hospitals (often travelling long distances on motorcycle in the heat and paying out of pocket for investigations like ultrasounds) because health functionaries were, in some cases, hesitant to enter the village due to the spread of COVID. For instance, the ANMs in West Singhbhum would call these pregnant women and give them a reminder to get vaccinated at the government hospital nearby but visited in person only once in a month. This reminder was only issued for immunisation, and not for any other services under the ANC umbrella after the unlocking process began towards early June in most of the districts under study.

In rare cases, during the lockdown, FLHWs guided and accompanied pregnant women to hospitals for health checks when these services were not being delivered at AWCs. Others reported not being able to access any healthcare services in pregnancy during the lockdown as they had contacted their FLHW at the beginning of the lockdown, who had told them that services were suspended and that she would intimate them when they resumed. Still others mentioned that their frantic calls to their community’s FLHWs, seeking clarity on the status of service delivery, had gone unanswered, which further contributed to a feeling of anxiety and hopelessness.

*“Now they (ANMs) say, “Sit a little far away”, they don’t give information properly. They give it in a hurry, and finish off in a hurry. We’re not able to avail benefits of services like earlier.”* (Pregnant Woman, District Lalitpur, Uttar Pradesh)

Some women, who normally visited private hospitals, found that services like ultrasounds were not available in these facilities during the lockdown, and hence were compelled to turn to government hospitals. These women were usually belonging to a relatively higher socio-economic group (compared to the majority of respondents), and may have felt that services delivered were of a higher quality in these private facilities. Forced to visit crowded and lower quality (in their perception) government hospitals during the pandemic, they reported feeling stressed.

Respondents felt that the sporadic frequency of service delivery by FLHWs was not adequate for them, and not being able to meet FLHWs in person limited the usefulness of their interaction. This may have been because those services that were perceived to be the most valuable (such as lab investigations like ultrasounds and blood tests) were not being administered, and in the case of those who could only contact FLHWs over the phone, even physical examination was not possible. Some pregnant women also reported not having adequate clarity on the timing of FLHWs’ availability in the village and being confused on whom to approach for guidance in their absence, which led to additional confusion and a feeling of alienation from the health system. They reported seeking information on their mobiles (via internet search) or contacting the ASHA over the phone for recommendations of medicines and care practices. Others were discouraged and lost faith in FLHWs, turning to others for assistance.

*“Anyway, those people (FLHWs) don't do anything, (and) they don't ask about our pregnancy, so we go to the hospital and ask the doctor.”* (Pregnant Woman, District West Singhbhum, Jharkhand)

Some pregnant women also reported not utilising ANC services even before COVID-induced lockdown (due to a variety of reasons such as not feeling they were valuable, not being permitted by their in-laws to attend, and so on), so they were not impacted in terms to a great degree by the lockdown.

### Plans for delivery

Nearly all the respondents reported a strong preference for institutional deliveries, owing to the supervision of the birth by trained medical personnel and access to vital information on caring for their newborn. They mentioned that by virtue of giving birth in the hospital, they would be able to receive important medication for themselves and their newborn if needed. Some even mentioned that delivering in a hospital brought the added advantage of helping them qualify for assistance under the government’s maternal health schemes.

*“If deliveries happen at home, where will we get information from (about the schemes and benefits)? When we go to the hospital, we will get information and services, and can take advantage of government schemes as well. (If we deliver at home), we won’t get anything.”* (Pregnant Woman, District Lalitpur, Uttar Pradesh)

Some respondents discussed the experiences of women who had delivered children in their community during the pandemic. All of these new mothers had reportedly opted for institutional delivery, and had not encountered any difficulties. Some differences were reported in the protocol followed at the onset of labour and during childbirth; only one family member was allowed to accompany the pregnant woman to the hospital, while there were no such restrictions before the pandemic. This resulted in anxieties for pregnant women as it was either the husband accompanying them in cases of lack of transport or ambulance to reach the hospitals without a female family companion, while in other cases, some other women family member was anticipated to accompany them whom they depicted signs of distrust in absence of the husband.

When at the hospitals, women in labour were kept further apart than was usual, and were discharged at the earliest possible time after delivery. When pregnant women responded to this, they showed some apprehensions as they were not sure if they would receive proper treatment and if the treatment they received would be sufficient. Some FLHWs even reported that pregnant women were mandated to be tested for COVID-19 prior to admission in the hospital for delivery, but this could not be corroborated by respondents.

*“It (deliveries) can’t happen like before. For example, earlier there used to be two to four sisters (nurses), who are not there now. They say that only one sister can do it (supervise the delivery).”* (Pregnant Woman, District West Singhbhum, Jharkhand)

When respondents (in particular, those who preferred institutional deliveries) spoke of their own plans for delivery in the thick of the COVID-19 pandemic, they expressed trepidation, since they were unsure of whether they would actually be able to give birth in the hospital and what the process would look like. This was due to their having had limited or no access to the regular course of institutional ANC services during their pregnancy and decreased FLHW interactions which meant they were not sure what to expect as they neared their delivery date.

*“My delivery will take place in a hospital but if this is not possible..(trails off). It may happen, it may not happen, we don’t know.”* (Pregnant Woman, District Chitrakoot, Uttar Pradesh)

Some pregnant women reported a fear of complications arising in delivery, while others were nervous about the process of childbirth because they did not know what steps to take if they started experiencing labour pains. This sentiment was echoed by even those pregnant women who had received check-ups and ultrasounds and were nearing their expected date of delivery. They claimed that since the start of the lockdown, all pregnant women in their locality had delivered in hospitals, not at home. Despite their fear of contracting the virus and confusion about the process, they still emphatically expressed a preference for delivery in health facilities over home birth.

However, the respondents were unable to articulate whom (FLHW) they planned to contact at the onset of labour, and how they planned to go to the hospital for delivery, given that they were apprehensive of using the ambulance. There was a deep sense of fear and confusion without any accompanying guidance on how to navigate the delivery. Additionally, due to the pandemic creating unprecedented conditions (such as constraints on mobility and limited access to healthcare functionaries and facilities), women could not look to their elders or community for guidance on managing their pregnancies, since they were also experiencing these conditions for the first time. For these reasons, pregnant women felt demoralised and confused, without any clear course of action.

## Discussion

The COVID-19 pandemic has irrevocably changed lives all over the world, and women’s experience of pregnancy and childbirth has been no exception. There are wide-ranging studies presenting the impact of COVID-19 situation on ANC service provision and access to services. Some of them bring to surface knowledge, risk perception, anxieties about service delivery, structural barriers aggravating the problem for pregnant women and FLHWs across the globe. In this setting, the behavioural barriers or enablers do play an important role. In this piece, we discuss how underlying behavioural principles played a part in supporting the components in the findings of this article. Behavioural principles affected both service provision by the healthcare system and utilisation of services by the end users; at times aggravating barriers to access maternal health services and influencing the planning of safe deliveries. These behaviour principles were further influenced by factors such as knowledge about the pandemic, trust in the health system and availability of resources.

There was a mixed reaction to COVID-19 among pregnant women. In our study on the impact of the pandemic and consequent lockdown on the lives of families in Uttar Pradesh, Madhya Pradesh and Jharkhand, we observed an overwhelming sense of fear and confusion. Across study areas, pregnant women expressed apprehension about availing of ANC services at health facilities due to fear of contracting COVID-19, as they believed it was dangerous for them and their unborn child. Though a small number of pregnant women did think of it to be not as dangerous as was purported, they did follow all the COVID-19 protocols to keep themselves away from the infection. All the participating pregnant women valued ANC visits, however they supported the closure of the health care centres and restricted themselves to homes in order to be safe. In many cases, pregnant women felt hapless and feared negative implications of lack of ANC services on the health and well-being of the fetus. Pregnant women felt especially vulnerable and anxious due to this half knowledge, i.e., while they knew that COVID-19 posed a special threat to them, they were left without any accompanying guidance on how to negotiate access to critical healthcare services during their pregnancy. This hampered the physical and mental well-being of pregnant women with very limited scope for help and hope.

At the time of the study, in the early months of the COVID-19 lockdown in India, the effects of the virusdisease on pregnant women were unknown, and hence there was no official guidance on the same. This lack of clarity may have heightened the anxieties of FLHWs, pregnant women and their families, since pregnancy is a time of uncertainty and high stakes as is. But pregnant women and their families adhered to what was told to them given their trust in mainly ASHAs. Whereas, the FLHWs weren’t comfortable spreading the information they had as they found themselves in situations that led to loss of confidence when trying to answer queries from the pregnant women, especially around the effect of COVID-19 on the fetus. This led FLHW to avoid such situations resulting in decreased interaction with the end-users. Furthermore, this restricted FLHWs’ decision-making ability and autonomy. This paved way to diminishing trust in the FLHWs by the end-users (despite their having known and trusted them for long), as the PWs sensed the discomfort within the FLHWs. This is an example of recency bias, wherein the end users favoured recent observations (i.e., FLHWs lacking in conclusive information or guidance with regard to COVID-19) over earlier observations (i.e., FLHWs being trusted, experienced healthcare providers for the community for a long time).

Evidently, pregnant women could see that the resources of the public healthcare machinery were dedicated to managing the impact of COVID-19 (and diverted from delivery of regular healthcare services), they could not see any positives resulting from this resource reallocation. This is an example of the behavioural principle of loss aversion, where people experience losses far more acutely than they experience gains, which in this case was the fact of minimal COVID-19 cases in the community due to quick deployment of frontline workers for community awareness.

Respondents felt as though the pandemic, and the myriad of protective measures it brought, created conditions for authority figures (like ANMs) to actively act on their prejudices based on caste and class, in the guise of enforcing social distancing guidelines. While in some cases caste-based discrimination may indeed have been meted out, in many cases it was simply presumed that social distancing guidelines were being enforced in line with the practice of untouchability. This was an instance of confirmation bias on the part of pregnant women, as they viewed real-life events (i.e. being asked to keep distance) as confirming pre-existing beliefs (i.e. the practice of untouchability).

As we understand from the findings, nearly all the pregnant women reported loss of income in the family given the pandemic and the lockdown that followed to curb it. This was one of the reported stressors for the family given that the special needs of the pregnant women and the plans around childbirth were disordered. Loss of income caused a lot more distress as compared to the fear of COVID-19. This had a direct impact on fulfilling the special needs of the pregnant women, medical aid and access to private health care. While in some districts, services like immunisation and basic physical examination were being delivered in a most basic, hurried manner, in others, there was no response from FLHWs despite repeated attempts to contact them. In the latter group, some pregnant women visited private facilities and district government hospitals depending on their financial resources, while others did not receive any antenatal care. Participants sensed uncertainties of the situation would lead to challenges in safe/institutional delivery and feared deprivation of maternal benefits provided at the public health services. Interestingly, the majority of respondents thought of institutional delivery in terms of the benefits it offered (i.e., gain framing) and not in the terms of the adverse outcomes it could help avoid (i.e., risk or loss aversion framing). Reduced interactions with the FLHWs left women without any support for planning childbirth including options of the hospitals and the means of transport. Having limited resources further made it uncertain for women to access private health care either. We observed that due to an absence of a clear directive or the expected schedule of contacts with health workers, there was a pessimistic outlook and a lack of initiative among pregnant women in terms of taking steps towards planning for delivery. This was indicative of an all-or-nothing, or absolutist mindset, a common cognitive distortion.

## Conclusion

As discussed in this paper, the COVID-19 pandemic created distress and anxiety in the community, particularly among pregnant women whose plans for delivery were thrown into disarray owing to the lockdown and negligible interface with frontline health workers. However, the most overwhelming cause of anxiety was to do with procuring adequate food for their families while their spouses’ livelihoods suffered, and not of contracting COVID-19. Since the pandemic was unprecedented, there was great uncertainty around when life would return to a state of normalcy. The pandemic also brought to the fore a mistrust among end users in the institutional healthcare system and its representatives, due to rapid spread of COVID-19 and high rates of fatality, unavailability of non-COVID-19 services, reports of discrimation suffered at the hands of frontline health workers, and proliferation of half-information on preventing and managing COVID-19. In order to bridge the gap, the pandemic situation has created, there is a need for a refurbishment of the healthcare system with focus on arranging undisrupted ANC services with dissemination of the correct information to the grassroots and enabling FLHWs with more autonomy for decisions during their interactions. This is warranted for the continued service delivery and sustained trust in the system by the end-users. The findings of this study will inform uptake of behavioural approach by stakeholders in ensuring that ANC services are available and accessible in times of crisis. However, there is more intervention-based evidence needed to provide a comprehensive understanding of barriers to ANC service provision to better inform behavioural strategies to address the problem.

**Table 1:** Inclusion criteria and distribution of the sample

Respondent category	Inclusion criteria	Sample size	Distribution	Number of units
Pregnant women	Women in the second or third trimester of pregnancy and accessing ANC services, irrespective of the number of previous pregnancies	12	Pregnant woman per village	12 Villages
Frontline health workers	Working FLHWs in each of the selected districts	17	1 Accredited Social Health Activist (ASHA), 1 Auxiliary Nurse Midwife (ANM) and 1 Anganwadi Worker (AWW) per district	6 Districts

## Data Availability

The datasets used and/or analysed during the current study are available from the corresponding author on reasonable request.
